# Improvement of *Torulaspora delbrueckii* Genome Annotation: Towards the Exploitation of Genomic Features of a Biotechnologically Relevant Yeast

**DOI:** 10.3390/jof7040287

**Published:** 2021-04-10

**Authors:** Carolina Santiago, Teresa Rito, Daniel Vieira, Ticiana Fernandes, Célia Pais, Maria João Sousa, Pedro Soares, Ricardo Franco-Duarte

**Affiliations:** 1 CBMA (Centre of Molecular and Environmental Biology), Department of Biology, University of Minho, 4710-057 Braga, Portugal; carolina.santiago.t@hotmail.com (C.S.); teresarito@med.uminho.pt (T.R.); jdanav@gmail.com (D.V.); tixand.tf@gmail.com (T.F.); cpais@bio.uminho.pt (C.P.); mjsousa@bio.uminho.pt (M.J.S.); pedrosoares@bio.uminho.pt (P.S.); 2 Institute of Science and Innovation for Bio-Sustainability (IB-S), University of Minho, 4710-057 Braga, Portugal; 3 Life and Health Sciences Research Institute (ICVS), School of Medicine, University of Minho, 4710-057 Braga, Portugal

**Keywords:** non-*Saccharomyces* yeasts, *Torulaspora delbrueckii*, fermentation, genomics, genome annotation

## Abstract

*Saccharomyces cerevisiae* is the most commonly used yeast in wine, beer, and bread fermentations. However, *Torulaspora delbrueckii* has attracted interest in recent years due to its properties, ranging from its ability to produce flavor- and aroma-enhanced wine to its ability to survive longer in frozen dough. In this work, publicly available genomes of *T. delbrueckii* were explored and their annotation was improved. A total of 32 proteins were additionally annotated for the first time in the type strain CBS1146, in comparison with the previous annotation available. In addition, the annotation of the remaining three *T. delbrueckii* strains was performed for the first time. eggNOG-mapper was used to perform the functional annotation of the deduced *T. delbrueckii* coding genes, offering insights into its biological significance, and revealing 24 clusters of orthologous groups (COGs), which were gathered in three main functional categories: information storage and processing (28% of the proteins), cellular processing and signaling (27%), and metabolism (23%). Small intraspecies variability was found when considering the functional annotation of the four available *T. delbrueckii* genomes. A comparative study was also conducted between the *T. delbrueckii* genome and those from 386 fungal species, revealing a high number of homologous genes with species from the *Zygotorulaspora* and *Zygosaccharomyces* genera, but also with *Lachancea* and *S. cerevisiae*. Lastly, the phylogenetic placement of *T. delbrueckii* was clarified using the core homologs that were found across 204 common protein sequences of 386 fungal species and strains.

## 1. Introduction

*Saccharomyces cerevisiae* is the yeast par excellence for both grape must and bread fermentations. Non-*Saccharomyces* species have long been associated with wine fermentation spoilage, mainly due to their negative sensorial traits and low fermentation abilities [1,2,3,4]. However, this paradigm has changed recently since positive effects, especially at the level of sensory attributes, have been reported for several non-*Saccharomyces* species [5,6,7,8,9,10,11]. *Torulaspora delbrueckii*, being genetically close to *S. cerevisiae*, has received significant attention for the benefits it brings to the wine and baking industries. In particular, regarding winemaking, *T. delbrueckii* is recognized for its improved aromatic complexity and mouthfeel properties [12,13,14]. Improvements in the aroma profiles are correlated with the production of specific fruity esters, thiols, and terpenes, and with low acetaldehyde levels, while mouthfeel properties are associated with the release of mannoproteins or polysaccharides that enhance sensory perception [14]. *T. delbrueckii*’s low acetic acid levels and elevated higher alcohols and glycerol production also positively alters the taste and the aroma [12,14,15,16,17]. One additional advantage of *T. delbrueckii* use in fermentation, in comparison with *S. cerevisiae*, is its higher capacity to produce C5 and C6 polyols [18], in particular D-arabitol, D-sorbitol, and D-mannitol, which allow for the physiological adaptation of yeast cells to stressful fermentative conditions, which positively influences its osmoadaptation and redox balance. However, certain disadvantages may come from the use of this yeast in wine fermentation, including the production of small amounts of 4-ethyl phenol, an increase in the concentration of precursors of some biogenic amines, such as histamine, or the fact that this yeast has been reported as being unable to complete the wine fermentation process by itself, which makes its use less economically viable than the current processes employing *S. cerevisiae* [19,20,21]. With this in mind, it becomes necessary to study the genetic, biochemical, and molecular features of *T. delbrueckii* in order to understand the optimal use of this yeast in wine fermentation, prompting possible biotechnological modifications and consequently obtaining maximum results regarding its aromatic and organoleptic parameters. Even though *T. delbrueckii* is not yet commonly used in the wine industry, several yeast companies already have different commercial strains available in their catalogs [14].

In the baking industry, *T. delbrueckii* is recognized as a superior choice due to its importance in the production of frozen dough products since it exhibits a very good baking ability and a high capacity to resist osmotic and freeze–thaw stresses [22,23,24]. In contrast, *S. cerevisiae*, the most commonly used yeast in the baking industry, loses viability fast under the same conditions [24]. This feature of *T. delbrueckii* is presumably related to its improved capacity to preserve its membrane integrity [25]. Similarly, in the brewing industry, *T. delbrueckii* has been studied in depth, showing relevant characteristics in beer fermentation, such as osmotolerance; resistance to weak acids, such as hop iso-α-acids production; low contribution of undesirable compounds, such as volatile phenols, acetic acid, and acetaldehyde [25,26,27,28]. In the cocoa bean industry, *T. delbrueckii* has shown impressive performances, enhancing the quality of the end product when used in cocoa bean fermentation. In particular, parameters such as the analytic profile and sensory perceptions of chocolate were altered, yielding a different aroma profile [29].

As suggested by the literature, a conjugation of *S. cerevisiae* and *T. delbrueckii* may be an option for improving wine quality by combining the advantages of both species [5,30,31,32,33]. Using protoplast fusion, Santos et al. [25] produced a hybrid strain (F1-11) of both species. F1-11 has improved resistance to acetic acid and ethanol, as well as a fructose consumption that is similar to *S. cerevisiae*. Beyond the ability to restart stuck fermentations, the results obtained with this strain were also of considerable importance regarding the improved flavor profile that was obtained.

Although *S. cerevisiae* is currently the most thoroughly annotated eukaryotic organism [34], as well as one of the most studied species, not much is known about the genome of *T. delbrueckii*. An in-depth study considering this yeast’s genome is needed in order to understand the genomic features underlying the advantages of its use. The genome of the *T. delbrueckii* type strain CBS1146 is organized into eight chromosomes and is described as being 9.52 Mb long with a GC content of 41.9% [35]. However, from the four genomes of *T. delbrueckii* strains that are available at the moment of this work (database assessed on January 2020)–strains CBS1146 [35], COFT1 [36], NRRL Y-50541 [37] and SRCM101298— only one, namely, COFT1, has non-nuclear genetic information that is publicly available and was sequenced using both short- and long-read technologies. Strain CBS1146’s genome was the first to be deposited, currently standing as an assembly at the chromosome level, on par with the NRRL Y-50541 strain. Finally, the genome of SRCM101298 strain, which was added to the database in July 2017, is considerably less explored and is assembled at the contig level. Additional genomes of *T. delbrueckii* were recently released [38]; however, not in the timeframe of the current work.

The objective of the present work was to explore the genomic data of *T. delbrueckii* that is accessible in public databases and to expand the annotation of this yeast’s genome, especially considering the strains whose genomes are scarcely annotated, namely, COFT1, NRRL Y-50541, and SRCM101298. With data obtained from the databases, a comparative study was conducted at a genomic level between this yeast and closely related species/genera, including *S. cerevisiae*, *T. globosa*, *Zygotorulaspora*, *Zygosaccharomyces*, and *Lachancea*. In addition, a phylogenetic analysis using the core homologs found across protein sequences was performed, comprising 386 fungal species and strains belonging to five fungal phyla, in order to clarify the phylogenetic placing of *T. delbrueckii* and to characterize this species’ phylogenetic relationships.

## 2. Materials and Methods

### 2.1. Genome Annotation

To perform the genome annotation, the four *T. delbrueckii* genomes available in the NCBI database (data collected in January 2020) were considered: CBS1146 (accession number GCA_000243375.1), COFT1 (GCA_003013175.1), NRRL Y-50541 (GCA_001029055.1), and SRCM101298 (GCA_002214845.1). The four genomes were downloaded from the NCBI and the Average Nucleotide Identity calculator (http://enve-omics.ce.gatech.edu/ accessed on 2 April 2021) was used in pairwise mode to establish comparisons between genomes in terms of their nucleotide content. The four genomes were then submitted to the Yeast Genome Annotation Pipeline (YGAP) [39] in order to establish potential coding regions in each of the *T. delbrueckii´*s chromosomes (Supplementary Data S1). After manually editing the YGAP output to correct the incongruencies, relevant information was extracted, in particular, the start and end positions of coding regions, strain orientation, and known homologs in the reference genome of *S. cerevisiae* (strain S288c). The potential coding regions (nucleotide sequences) reported by YGAP were extracted from the complete *T. delbrueckii* genome into a FASTA file. Assessment of the genomes’ completeness was performed using BUSCO (Benchmarking Universal Single-Copy Orthologs )software, version 5.0 [40].

Proteins identified by YGAP as having no homology with *S. cerevisiae* were scrutinized using BLAST (Basic Local Alignment Search Tool; E-value cutoff of 10^−6^) against the NCBI Reference Sequence (RefSeq) collection (ncbi.nlm.nih.gov/refseq/), and the results were combined by assessing the number of taxonomic correspondences (top hits) for each protein.

Functional genomic annotation was performed with eggNOG-mapper v.5.0 [41] by considering proteins predicted by YGAP and choosing only orthologs that were inferred from the experimental evidence. The results were described considering Gene Ontology (GO) terms, Kyoto Encyclopedia of Genes and Genomes (KEGG) pathways [42], and clusters of orthologous groups (COGs) with their associated functional categories [43].

### 2.2. Homology Analysis

A BLASTP analysis was performed using the aforementioned FASTA files as queries against a local database of 386 fungi containing non-redundant sequences and considering only one representative organism of each fungal species, with the exception of some species that are closely related to *T. delbrueckii* that had more than one strain. An E-value cutoff of 10^−6^ was considered to exclude false results. In particular, from the group of 386 organisms, 19 strains of *Torulaspora*, *Zygotorulaspora*, and *Zygosaccharomyces* genera were also annotated using YGAP in order to allow for their inclusion to search for homologies with *T. delbrueckii* (results summarized in Supplementary Table S2). Thirty-five *S. cerevisiae* strains whose origins were related to winemaking or fermentative beverages were also included in the homology analysis. The *T. delbrueckii* COFT1 genome was selected as the query since it was the only one with non-nuclear information (mtDNA sequence was also available), it had a complete genome assembly, it was sequenced using both short and long-read technologies, and it corresponded to a strain originating from winemaking environments [36], following the objectives of the present work. For comparison, we also considered the three remaining *T. delbrueckii* assemblies. In summary, in the BLAST analysis, each query corresponded to the alignment of a protein-coding sequence in *T. delbrueckii* COFT1 against the local database.

### 2.3. Phylogenetic Analysis

The full proteome of *T. delbrueckii* COFT1 was used to search the common proteome portion of 386 fungi in a total of 334 fungal species’ defined proteomes belonging to five phyla: Ascomycota, Basidiomycota, Chytridiomycota, Mucoromycota, and Microsporidia. The taxonomic classification of each species was performed according to Mycobank (mycobank.org/; accessed on January 2020). Proteins of *T. delbrueckii* COFT1 were used as BLAST queries in a local database containing the proteins of the other organisms. Following the BLAST searches, we filtered the proteins where representatives of the other 385 organisms were detected. When more than one homolog was present per species, in order to avoid any bias, one was selected randomly. However, this situation was extremely rare among the obtained proteins.

Each set of probable homologous proteins (containing the query and the results obtained for that query) were multiple-aligned using Clustal Omega (v.1.2.2; http://www.clustal.org/omega accessed on 2 April 2021) [44]. Following the alignments, all proteins from a given species were concatenated using the alignment results. With this approach, we obtained the common proteome between the analyzed organisms (a core conserved proteome containing mostly essential genes not related to specific biological traits of each species) that were fully aligned.

The concatenated alignment was used for the phylogenetic reconstruction by considering the maximum likelihood in IQ-TREE (http://www.iqtree.org/ accessed on 2 April 2021) [45] with the JTT model of amino acid evolution and gamma-distributed rates (four rates) with 500 bootstrap replicates. Figtree (http://tree.bio.ed.ac.uk/software/figtree accessed on 2 April 2021) was used to visualize and edit the tree.

## 3. Results and Discussion

### 3.1. Torulaspora Delbrueckii Genome Annotation

The genome annotation of *T. delbrueckii* using YGAP yielded between 4361 and 5016 putative coding sequences (CDSs), as described in Table 1. In particular, the lowest number of CDSs was obtained when considering strain NRRL Y-50541, even though it was the longest genome of the four considered (11.53 Mb, in comparison with an average of 9.42 Mb of the remaining three). This fact was mainly due to the existence of long sequences of “N” bases that were inserted to establish links between neighboring contigs in this genome, which also explains the lowest BUSCO genome completeness score (Table 1) and the relevant differences found when comparing with the remaining three strains’ genomes (Table 2). In detail, only a 97.98% similarity was found when comparing this strain’s genome with the genome of the type strain CBS1146. The highest number of CDSs was obtained when considering the genome of strain SRCM101298 (5016).

The genome of strain CBS1146 already had a good level of genome annotation available in the NCBI [35]. However, the reannotation of this genome using the updated version of the YGAP pipeline yielded a slightly higher number of CDSs (4978) in comparison with the 4970 described in NCBI’s version. In detail, from the 4878 CDSs, a total of 429 did not match the previous annotation of Gordon et al. [35]. A closer examination of these genes revealed that 397 had a direct correspondence with genes from the previous annotation, most often with only one altered coordinate. In 250 cases of the 397 pairs, our annotation suggested an extended gene in relation to the previous one. It is important to point out that in the analysis of these 397 pairs, the inferred function and homology profiles were the same in both annotations. A list of the gene pairs with coordinates from both annotations is indicated in Supplementary Data S3. Thirty-two genes were unique to our annotation (Supplementary Data S4), while 21 were only present in the previous annotation. While a few of these were likely false positives, as no homology or low values of homology with genes from other fungi were detected, at least four showed relevant homology with *S. cerevisiae* S288c and other species and should be considered. As such, we present the list of those 21 genes as a possible complement to our annotation (Supplementary Data S5).

A high homology was found by YGAP between *T. delbrueckii* genomes and the one of *S. cerevisiae* S288c (Table 1), with an average value of 4352 protein-coding sequences detected as homologous, corresponding to 90.52% of the total annotated genes. The highest number of homologies with this strain (4514) was obtained with the genome of strain CBS1146, i.e., the *T. delbrueckii* type strain, even though this number was very similar to the one obtained with COFT1 (4506) and with SRCM101298 (4513).

**Figure 1:**
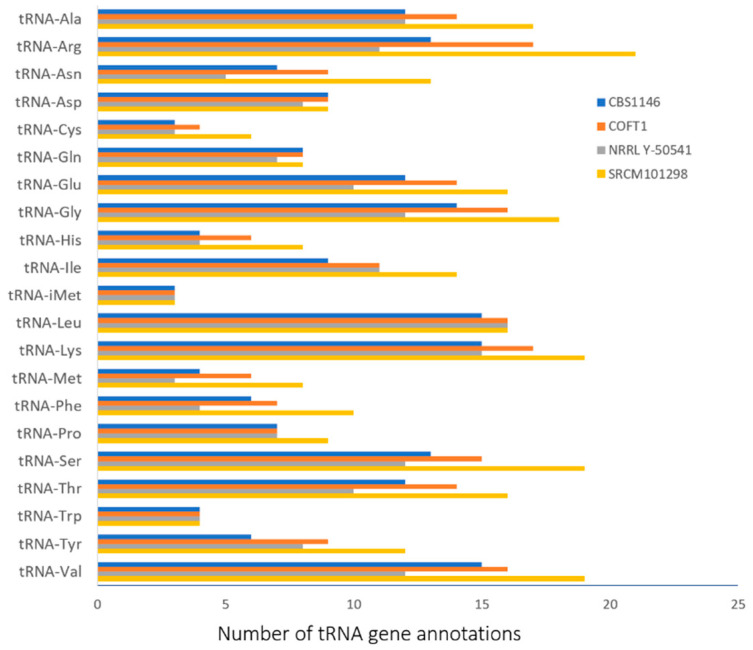
Total number of tRNA gene annotations obtained with the YGAP pipeline for the four *Torulaspora delbrueckii* strains.

In addition, the total number of transfer RNA (tRNA) genes predicted by YGAP was compared between the strains (Table 1 and Figure 1). The results show a higher number of almost all types of tRNA in the genome of strain SRCM101298, which was not directly related to the total number of CDSs or with the genome size. These differences obtained in the annotation of tRNAs could explain the slight variation observed in the average nucleotide identity comparisons of Table 2. Nevertheless, the combined results of Table 1 and Table 2 show that the genomes of the three strains, excluding NRRL Y-50541, showed a high level of genome completeness, high level of similarity, and were annotated with high robustness with the present pipeline. Strain NRRL Y-50541 showed a smaller number of the annotated tRNA, which was expected due to the quality problems associated with this genome, as already discussed.

**Figure 2:**
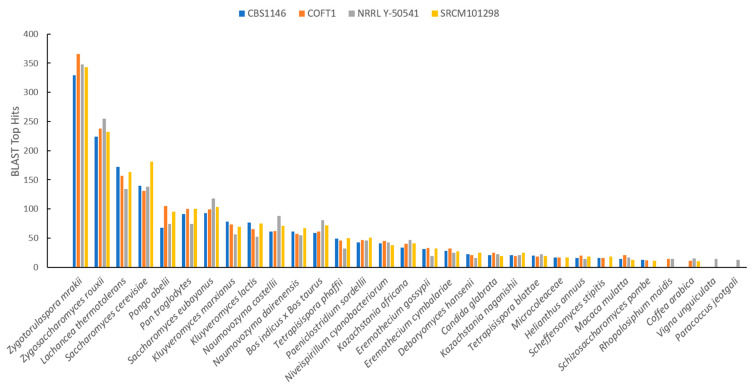
Basic Local Alignment Search Tool (BLAST) top hits distributed by species based on the best sequence alignments and lowest E-values by considering the proteins that were not identified by YGAP in the four *T. delbrueckii* strains and the five top hits for each protein. Only species with more than 20 top hits are shown.

Furthermore, BLAST was used to identify proteins that revealed no homology with *S. cerevisiae* S288c, and, in this way, were labeled as unidentified. In detail, the unidentified proteins (between 464 and 503, as shown in the last column of Table 1) were used as a query against the NCBI RefSeq database, and BLAST results (top 5 hits for each protein) were clustered by considering the taxonomic groups with the top results. Figure 2 summarizes the obtained results, including only those species with more than 20 hits. Since these proteins were rare across the database, leading to various spurious results regarding organisms in the top 5 matches, all results were confirmed by using a reciprocal BLAST to test for the presence of *T. delbrueckii* in the matches when using these proteins as queries. Only those where a positive match was obtained were maintained in the analysis. These eliminated genes that were almost certainly not realistic matches but, instead, they were random matches when homologous genes were not detected in fungi (or at least not detected outside the main matches in *Zygotorulaspora* and *Zygosaccharomyces*), which, additionally, was supported by no matches detected in BLAST against a fungi database in the following analysis.

The results showed that the higher percentage of unidentified proteins had a match with species *Zygotorulaspora mrakii*, as expected, followed by the genera *Zygosaccharomyces* and *Lachancea*. Furthermore, between 130 and 177 matches with *S. cerevisiae* were found, corresponding to proteins that were not detected by YGAP as having homology with *T. delbrueckii.* Our approach allowed for addressing and characterizing *T. delbrueckii*’s full proteome for the first time, providing an important foundation for further studies exploring biotechnological uses of this species.

### 3.2. Functional Annotation

**Figure 3:**
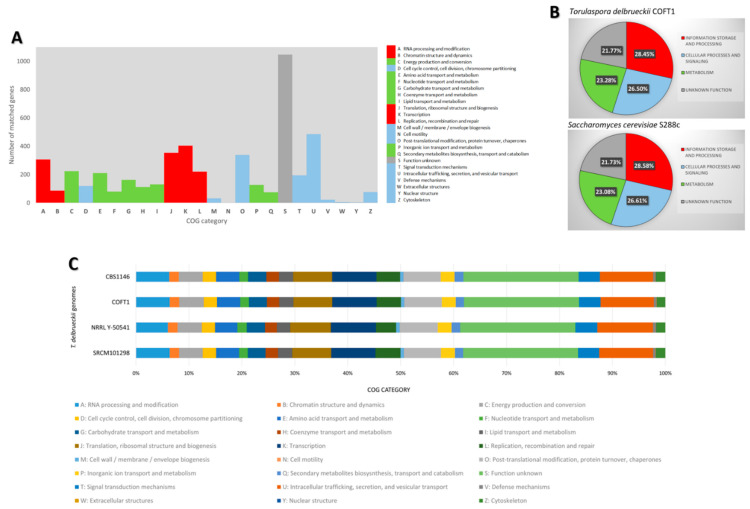
eggNOG classifications of the annotated *T. delbrueckii* genes. The functional annotations were divided into 24 categories, corresponding to clusters of orthologous groups (COGs). (**A**) Number of genes grouped in each of the 24 COG categories for the *T. delbrueckii* COFT1 genome. The colors are indicative of the functional categories used in panel B. (**B**) Classification of the genes into functional categories, comparing *T. delbrueckii* COFT1 with the reference *S. cerevisiae* S288c. (**C**) Comparison between the four available genomes of *T. delbrueckii* in terms of the number of grouped genes (as a percentage) in each COG category.

eggNOG-mapper was used to perform functional annotation for the deduced *T. delbrueckii* proteins, offering insights into its biological significance (Figure 3). A total of 4814 genes of the *T. delbrueckii* COFT1 genome (96.1% of the total annotated genes) were clustered using eggNOG-mapper in 24 COGs (Figure 3A), which were gathered in three main functional categories, as shown in Figure 3B. The results show that, for this strain, the higher percentage of annotated genes were related to “information storage and processing” (28.45%) and “cellular processes and signaling” (26.50%). A high number of annotated genes did not have a clear function attributed by egg-NOG (21.77%), and 23.28% of the genes were related to “metabolism.” The most abundant COG category with a function attributed in the genome of *T. delbrueckii* COFT1 was “intracellular trafficking, secretion, and vesicular transport” (485 genes, corresponding to 10.1% of the annotated genes), followed by “transcription” (404/8.4%). The least abundant categories were “cell motility” with only one associated gene (0.02%), and “nuclear structure” with three (0.06%).

A comparison between *T. delbrueckii* COFT1 and *S. cerevisiae* S288c regarding the general functional categories displayed in Figure 3B revealed a slightly smaller percentage of genes of *T. delbrueckii* strain associated with “information storage and processing” and “cellular processes and signaling” categories, relative to the *S. cerevisiae* values. Interestingly, a slightly higher percentage of genes in *T. delbrueckii* was revealed to be related to the “metabolism” (23.28%) category, in comparison with the percentage of *S. cerevisiae* genes associated with this category (23.08%). Analysis of this result revealed that “energy production and conversion” grouped 224 genes of *T. delbrueckii* and only 217 of *S. cerevisiae*. Observed differences were related with the *T. delbrueckii* COFT1 gene being annotated as associated with the “Acetamidase/Formamidase family,” “ATP-hydrolysis-coupled proton transport” (similar to *S. cerevisiae* atp9 and atp6 but absent in the S288c eggNOG annotation), “LDH MDH superfamily,” and “cytochrome-c oxidase activity” (in addition to the COX20 and COX6 identified in S288c). Although these differences were not enough to explain the aroma differences in the fermentations performed by *T. delbrueckii*, they could be associated with differences in the fermentative performance that is commonly associated with *T. delbrueckii* and should be further investigated. However, this information must be analyzed with caution since by not being a technological strain, comparisons with S288c should not be extended to the *S. cerevisiae* species level.

Small intraspecies variability was found when considering the functional annotation of the four *T. delbrueckii* available genomes (Figure 3C). Particular differences were observed, especially regarding the genome of strain NRRL Y-50541 and the COG categories “L: replication, recombination, and repair” and “I: lipid transport and metabolism,” for which a decrease in the number of genes in those groups was detected (149 and 98 genes, in comparison with 220 and 130 annotated in COFT1, respectively). However, due to the lack of the completeness of this genome, as discussed, these differences should be analyzed with care.

A detailed comparison between strains COFT1, CBS1146, and SRCM101298 is available in Supplementary Data S6, showing 158 triple-wise differences between the three strains with reference to the 4814 eggNOG annotated genes. The great majority of variability was related to the duplication of some genes in some strains, and with the fact that COFT1 also has a mitogenome included in the assessed genome. However, some other key differences need to be highlighted: (i) Two genes were annotated in the COFT1 genome as being associated with “ATP-synthesis-coupled proton transport,” and one gene with “electron transport coupled proton transport,” with this annotation being absent from the remaining two strains. In addition, regarding the differences found when compared with the *S. cerevisiae* annotation, these results highlight an increased annotation of COFT1 strain’s proteins to functions related to proton transport. (ii) A lack in the genome of strain CBS1146 of a gene homologous to the YDR287W of *S. cerevisiae*, which is involved in the biosynthesis of inositol, which is important in low-temperature fermentations; this can be explained by the environment associated with this yeast, probably away from wine fermentations. (iii) The absence in strain SRCM101298 of a gene that is homologous to BFR2 of *S. cerevisiae*, which is related to the resistance to antiviral Brefeldin A and is known to have its expression induced during the lag phase and by cold shock.

The COG database [46] has been a popular database for the functional annotation of microbial genomes, allowing for the reliable assignment of orthologs to most genes [43]. Orthologous genes are products of speciation, and when clearly defined, they allow for outlining relationships between species and to understand their evolution. The identification of orthologous genes was previously used to successfully identify differences and similarities between species, annotating their functional genetic information, and proposing functions in newly sequenced genomes [47]. KEGG [42,48] was used in this work to scrutinize the eggNOG results by interpreting the biological function of genes via the interpretation of enzymes and biochemical processes. In the present study, eggNOG-mapper allowed for organizing the 4814 genes in 3123 KEGG Orthology annotations (Supplementary Data S7). Of note is the fact that several genes were assigned to KEGG Orthology groups related to “transporters,” corroborating the previous division into COG categories, and the detailed differences highlighted in strain COFT1. The knowledge about relevant aspects of biology and biochemistry is still limited regarding *T. delbrueckii*, including details about the transport mechanisms and transporter collection, for example, to assure the uptake of sugars during fermentation. In *S. cerevisiae*, these transporters have a key role in the metabolism of carbon compounds [49,50], and could be based on the adaptation to new environments, allowing for the fermentation of new carbon sources [51]. Regarding *T. delbrueckii*, recent results show a similar relevance attributed to transporters [52,53], stating the importance of this species for the food industry.

### 3.3. Homology Analysis

**Figure 4:**
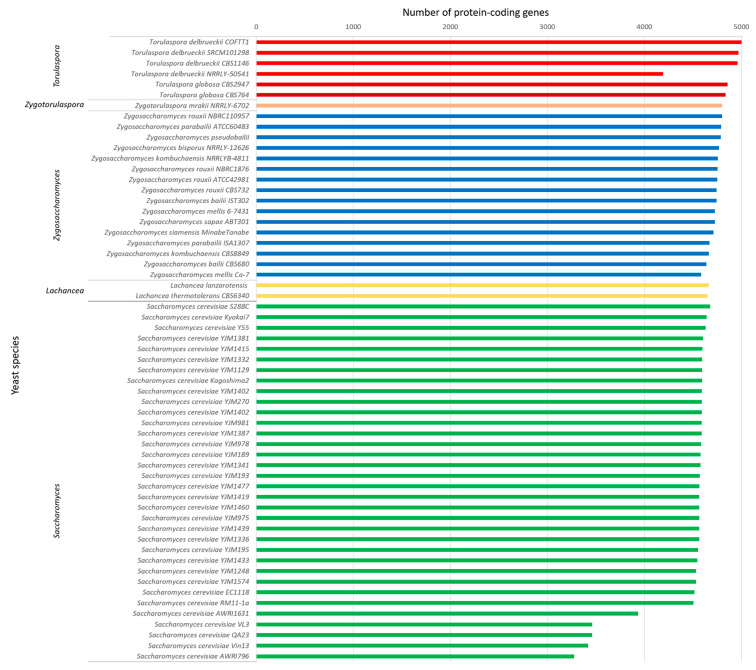
Homology comparison between the protein-coding genes of *Torulaspora delbrueckii* COFT1 genome (used as the reference) and 56 related yeast species/strains. The protein-coding regions of the COFT1 genome were detected using YGAP and the homology was determined using a BLAST analysis. Only species/strains with more than 4600 homologs are presented (with the exception of some *S. cerevisiae* strains and *T. delbrueckii* NRRL Y-50541, which were included for comparison); the results for the complete set of 386 yeasts are available in Supplementary Data S8.

Upon obtaining and parsing the output from YGAP, as described in the Materials and Methods section, a BLAST analysis was performed to search for homologies between the selected coding regions of *T. delbrueckii* and the NCBI genome database (database assessed in August 2020) such that putative matches could be considered as homologous. Comparisons were analyzed by considering 386 yeasts with full genome sequences available in the NCBI. From the 386 genomes, 329 corresponded to different species, and the remaining 57 corresponded to different strains of some species that were previously known to be closely related with *T. delbrueckii*; these 386 genomes were used to obtain greater detail in the analysis and detect the proximity to specific strains. The results (Supplementary Data S8) show the number of homologous protein-coding sequences obtained for all the organisms. Figure 4 summarizes the main results regarding the species and genera with the highest number of detected sequences that were homologous with *T. delbrueckii* from the genera *Zygotorulaspora*, *Zygosaccharomyces*, *Lachancea*, and *Saccharomyces*.

Strain *T. delbrueckii* SRCM101298, originating from fermented food, presented the higher percentage of homology with COFT1 (4969 common protein-coding sequences out of 5009 used as the query, corresponding to 99.2%) inside the group of *T. delbrueckii* strains, although it was very close to the one obtained for the type strain CBS1146 (99.0%, 4960 common coding sequences). The three *T. delbrueckii* genomes shared 4960 homologous sequences out of a total of 5009 putative coding sequences. The fourth genome considered, namely, *T. delbrueckii* NRRL Y-50541, revealed lower homology, a fact that was in line with differences already found when analyzing FASTA files obtained from YGAP. We believe that these differences must not have been due to true differences but, instead, to the overall quality of the assembled genome that led to an absence of some parts of the genome. As stated before, this genome contained large sequences of “N” bases that were inserted to establish links between contigs, which led to a low number of predicted genes by YGAP.

When comparing *T. delbrueckii* with other species, the highest number of homologous sequences was detected in comparison with the genomes of *T. globosa*, as expected since they share the same genus. Strain *T. globosa* CBS2947 shared 4858 coding sequences with the genome of *T. delbrueckii* COFT1, corresponding to 97.0% of the genes, while a total of 96.6% of homologous genes was detected with strain *T. globosa* CBS764. On average, the genera *Zygotorulaspora* and *Zygosaccharomyces* revealed 95.8% (4799 sequences) and 94.3% (4725 sequences) of homologous sequences with *T. delbrueckii*, respectively. In particular, for the *Zygosaccharomyces* species, between 4582 and 4800 putative coding sequences were revealed as homologous with *T. delbrueckii* COFT1, with *Zygosaccharomyces rouxii* NBRC110957 being the most homologous strain (95.8% of coding sequences). Furthermore, two species of *Lachancea* genus— *Lachancea thermotolerans* and *Lachancea lanzarotensis*—showed a relevant amount of homologous genes with *T. delbrueckii* COFT1—92.8 and 93.1%, respectively—which is in accordance with their role in fermentation, especially in fruit wine fermentation [54]. Surprisingly, the genus *Saccharomyces*, in particular the species *S. cerevisiae*, revealed slightly less homology with *Torulaspora* than the *Zygosaccharomyces* species. In fact, almost all *S. cerevisiae* strains (strain S288c was the only exception) revealed a smaller number of homologous genes (between 4506 and 4642 coding sequences) with the *T. delbrueckii* genome than those obtained by the great majority of *Zygosaccharomyces* species.

Within the group of *Saccharomyces* species, the *S. cerevisiae* reference strain S288c showed the highest percentage of homologous genes with the genome of *T. delbrueckii* COFT1, with 4676 homologous sequences (corresponding to 93.5% of the coding sequences). Regarding other strains related to winemaking, from which a higher homology would hypothetically be expected since *T. delbrueckii* COFT1 originated from winemaking environments [36], this value was even smaller than the one obtained for the laboratory strain S288c. The lowest number of homologous putative coding sequences was obtained for the Australian strain AWRI796 (3276–65.5%), which is used worldwide as a commercial strain for winemaking (Mauri Yeast, Australia). Previous studies revealed that this industrial strain, although showing a good fermentation performance, has particular genomic profiles that are mainly related to its extremely high sensitivity to harsh and stressful enological conditions [55], which could explain its distance from other winemaking strains considered in the present study. In fact, this connection between genetic features and their relevance in phenotypic variability and applicability in winemaking was shown before for 172 other *S. cerevisiae* wine strains [56,57]. *Saccharomyces eubayanus* and *Saccharomyces paradoxus* revealed similar levels of homology to the average one obtained with *S. cerevisiae—*91.5% and 92.8%, respectively.

Genera *Naumovozyma, Kluyveromyces*, *Kazachstania*, and *Tetrapisispora* followed in terms of the decreasing number of homologous genes with *T. delbrueckii*, with a smaller number of sequences obtained on average (4580, 4517, 4531, and 4469, respectively). One case of particular notice was related to the genome of *Candida glabrata*, which showed a total of 4492 hits (89.7%), which is a value that is similar to those obtained by *S. cerevisiae* and different from the ones obtained by other *Candida* species, which points to possible proximity in genomic terms between these two species. This fact was also observed when the full yeast’s proteome was analyzed, as will be shown and discussed below.

Several studies have compared the fermentative potential of *S. cerevisiae* with that of *T. delbrueckii*, although a full genomic comparison is still lacking, mainly due to the fact that some of *T. delbrueckii*´s available genomes are still sparsely annotated, in particular, the ones of strains COFT1, NRRL Y-50541, and SRCM101298; as such, they could not be easily compared with the well-annotated genome of *S. cerevisiae*. Although having marked differences regarding producing secondary metabolites during fermentation, as well as resisting stresses, large similarities have been found at taxonomic and genetic levels between the two species. However, care is needed when comparing both species, mainly strain S288c, because this strain is not a technological one. Therefore, associations of differences between the genomes of *T. delbrueckii* and *S. cerevisiae* S288c should not be generalized to differences with *S. cerevisiae*. In the present study, a total of 93.4% of the homologous sequences were detected between the genomes of *T. delbrueckii* COFT1 and *S. cerevisiae* S288c (Figure 4). Even though a higher score was expected due to the recognized proximity between the *Torulaspora* and *Saccharomyces* genera, it is not surprising since the *Saccharomyces* genus has evolved after a genome duplication event, in contrast with the *Torulaspora* and *Zygosaccharomyces* genera [58,59]. The comparison between *S. cerevisiae* and *T. delbrueckii* has been long discussed, and regarding wine fermentation, *T. delbrueckii* mainly shows great potential to serve as an alternative to *S. cerevisiae* due to its capacity to produce a different array of secondary metabolites. A previous comparative analysis of the transcriptome and metabolome of both species [17] detailed some important differences, in particular, the lack of multiple genes in *T. delbrueckii*, highlighting differences in the glycolic and fermentation pathways, together with a conclusion about the lower volatile acidity associated with *T. delbrueckii*.

The homology was somewhat higher when the genome of *T. delbrueckii* was compared with the available genomes of the *Zygosaccharomyces* species (between 91.5 and 95.8% of homologous detected; Figure 4). This percentage seems to indicate proximity between *T. delbrueckii* and *Zygosaccharomyces* species that were higher than the one found when comparing with *S. cerevisiae* in terms of the genomic analysis. However, by not being markedly different, the similarity pointed to the proximity between the three genera, a fact that has already been extensively discussed before, especially regarding physiological properties, but also using some genetic segments [17,60,61,62]. In detail, using multigene sequence analysis, Kurtzman and Robnett [63] compared 75 species belonging to the “*Saccharomyces* complex,” including species of *Saccharomyces*, *Torulaspora*, and *Zygosaccharomyces*. The species were divided into 14 clades, with the species of genera *Torulaspora* and *Zygosaccharomyces* being placed into three mixed groups (7, 8, and 9), apart from the *Saccharomyces* species (both sensu stricto and sensu lato). To clarify these mixed clusters, authors proposed the creation of a new genus in 2003, namely, *Zygotorulaspora*, comprising the species *Zygotorulaspora florentinus* and *Zygotorulaspora mrakii* [64]. Our results are in line with the conclusions of their work since *Zygotorulaspora mrakii* NRRL Y-6702 showed 95.8% of sequences to be homologous with *T. delbrueckii*, a value higher than the one obtained for the genera *Zygosaccharomyces* and *Saccharomyces.*

Furthermore, of particular interest was the homology detected for the genus *Lachancea*: 93.1% of genes were found to be homologous between *T. delbrueckii* COFT1 and species *L. lanzarotensis* and 92.8% with species *L. thermotolerans*. This proximity between *Lachancea* and *T. delbrueckii* was shown before, detailing shared traits that were mainly related with osmotolerance and ethanol resistance [25,65], but also remarkable similarity considering the clusters of the mating-type switching endonuclease HO genes [38]. *L. thermotolerans,* especially, has been associated with a strain-dependent production of a diverse range of metabolic intermediates for L-lactic acid production [65,66], as well as ethyl lactate [67]. The high homology detected between the two species in the present study is also in line with their common capacity to ferment maltose, producing significant amounts of acetyl esters and long-chain ethyl esters [68], pointing in this way to the potential use of *T. delbrueckii* for industrial beer fermentation.

### 3.4. Phylogenetic Analysis

**Figure 5:**
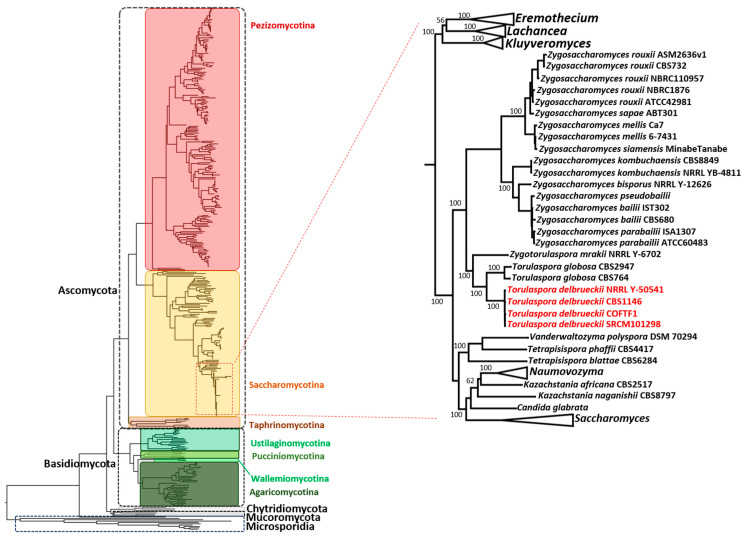
Phylogeny of fungi regarding 386 fungal core genomes (alignment of 204 common proteins). The phyla are highlighted using dashed lines and the subphyla are identified using colored boxes. The placements of the *Torulaspora delbrueckii* strains are shown in detail relative to the closely related species inside the Saccharomycotina subphylum. The concatenated alignment was used for the phylogenetic reconstruction by considering the maximum likelihood and 500 bootstrap replicates. The bootstrap values (in percentages) are shown for the highlighted branches. A detailed phylogenetic tree and the full bootstrap values are available in Supplementary Data S10.

A local database was compiled using 386 defined fungal proteomes and compared against *T. delbrueckii* COFT1. Only yeasts with a fully characterized and annotated proteome were considered (database built in August 2020). The entire proteome of *T. delbrueckii* COFT1 (5009 proteins) was used in a BLAST analysis against the database and a total of 204 *T. delbrueckii* proteins (Supplementary Data S9) displayed homologs in the 386 fungi. A phylogenetic analysis (Figure 5) was performed by considering the alignment of the core concatenated proteins present in the 386 organisms.

Our results display a general evolutionary relationship between strains independent of specific physiological adaptations of the species. To our knowledge, it is the first time that this analysis has been performed in terms of considering such a high number of organisms and assessing their common proteome. Even though many of the phylogenetic relations were already known, our results increased the robustness of several yeast species’ placement by assessing the core group of 204 common proteins, and allowed for separating the five phyla of fungi–Ascomycota, Basidiomycota, Chytridiomycota, Microsporidia, and Mucoromycota–with high branch support (the full bootstrap values are available in Supplementary Data S10). The Microsporidia phylum showed a marked distance from the remaining four, representing a deeper split within the fungi group, only with the species *Mitosporidium daphnia* showing proximity to the four phyla. Considering the fungal subdivisions, the core proteome revealed a clear distinction between the seven taxa of Ascomycota and Basidiomycota (colored boxes in Figure 5). This group of core proteins appeared to be conserved across all fungal species, even considering the ones that were more phylogenetically distant, for example, the four species of genus *Encephalitozoon*— *Encephalitozoon romaleae*, *Encephalitozoon hellem*, *Encephalitozoon intestinalis*, and *Encephalitozoon cuniculi*—belonging to class Microsporidia, and being the most common pathogenic genus against humans and domesticated animals in this class (Supplementary Data S10). Importantly, all phyla and major taxonomic groups within the phyla established monophyletic clades, which attested to the robustness of the tree.

A more detailed analysis of the *T. delbrueckii* placement (highlighted in detail in Figure 5), confirmed the species as phylogenetically closer to the *Zygosaccharomyces* species than to *S. cerevisiae*, as shown in our previous analysis. Our results are in accordance with the work of Shen et al. [69], showing the phylogenetic reconstruction of more than 300 budding yeasts, but allowing for a robust elucidation (bootstrap value of 100%) of the phylogenetic placement of the *Torulaspora* branch, which is one of the branches that was concluded as not being robustly recovered in the work of Shen et al. Our results highlighted some genetic distance between the *Torulaspora* and *Saccharomyces* genera in favor of other genetically closer genera, such as *Zygosaccharomyces* and *Zygotorulaspora*, representing an advancement of knowledge, especially in terms of assessing the high branch support obtained with the core proteins.

When analyzing the phylogenetic tree of Figure 5, it was clear that the two *Z. rouxii* strains were the most closely related, and then grouping with a clade containing two *Zygosaccharomyces parabailii* and two *Zygosaccharomyces bailii* proteomes, with the *Zygosaccharomyces* genus clearly being the second-most closely related with *T. delbrueckii*, just behind the *Zygotorulaspora* genus, which is considered as a sister genus. The *S. cerevisiae* strains appeared only further away in a group composed of other species from the genera *Kazachstania*, *Naumovozyma*, *Tetrapisispora*, and *Vanderwaltozyma*. Importantly, and contrary to what was concluded in the homology analysis, *Lachancea* spp. were located in a separated branch to the one containing *Saccharomyces* and *Torulaspora* species. The results showed that, although *Saccharomyces* and *Torulaspora* species were evolutionarily closer, *Lachancea* and *Torulaspora* had a higher biochemical and physiological proximity, as shown by the higher number of homologous genes, as already discussed.

Furthermore, of notice is the fact that *C. glabrata* was located in the *Saccharomyces* group, close to *S. cerevisiae* wine strains and to *S. paradoxus* and *S. eubayanus*, and apart from the *Candida* subclade. This result is in accordance with what was shown before [70], regarding the similarity between *C. glabrata* and *S. cerevisiae*, although the first has evolved to acquire pathogenicity in mammalian hosts.

## 4. Conclusions

*Torulaspora delbrueckii* is a non-*Saccharomyces* yeast that has been referred to many times as an alternative to *Saccharomyces cerevisiae*, especially in wine and bread fermentation since it contributes a novel palette of aroma and flavor characteristics to the final product. The basis of this novelty has largely been searched, and genomic fingerprints of *T. delbrueckii* that are exclusively found in this species are believed to be interconnected with this question. However, the genome of *T. delbrueckii* is still sparsely annotated, especially considering the majority of available genomes, and in comparison with the perfectly annotated genome of *S. cerevisiae*; this does not allow for us to draw conclusions about the particularities of this fermentative yeast. The present work represents a successful effort to increase and improve the annotation of *T. delbrueckii*´s genome, identifying homology between this yeast and hundreds of other fungal species, together with providing a functional annotation of their coding genes, increasing their biological significance. The differences detailed in this work when comparing *T. delbrueckii* strains with several yeast species with known biotechnological importance provide an opportunity to exploit new biotechnological applications or the combined utilization of several yeast species to enhance fermentative traits. In particular, our functional annotation revealed some particular differences between *T. delbrueckii* and *S. cerevisiae* strain S288c that were mainly related to the “metabolism” category and with other categories related to fermentative performance. These results support the growing interest in *T. delbrueckii* strains, unraveling the diversity of potential biotechnological applications of this species. In the future, the proposed pipeline for genome annotation should be employed to annotate the genome of other *T. delbrueckii* isolates, which could be of high importance when selecting strains with very particular characteristics.

## Figures and Tables

**Table 1:** *Torulaspora delbrueckii* genomes used in this study and the corresponding number of protein-coding sequence (CDS) transposable elements and transfer RNA (tRNA) genes predicted by Yeast Genome Annotation Pipeline (YGAP).

Strain	Source	Reference	Genome Size	BUSCO Genome Completeness (%)	Coding Sequences	Transposable Elements (TY)	tRNA Genes	Homologies with *S. cerevisiae*	Unidentified Coding Sequences ^1^
CBS1146	Unknown; type strain	[35]	9.22 Mb	98.5	4978	5	191	4514	464
COFT1	Wine fermentations	[36]	9.36 Mb	98.1	5009	5	223	4506	503
NRRL Y-50541	Mezcal-fermentations	[37]	11.53 Mb	80.2	4361	6	180	3875	486
SRCM101298	Fermented food	-	9.68 Mb	98.2	5016	7	274	4513	503

^1^ Corresponding to no homologies with *S. cerevisiae* S288c detected by YGAP.

**Table 2:** Nucleotide identity matrix. The values represent the average amounts of pairwise differences (as a percentage) obtained by comparing the four *T. delbrueckii* genomes assessed in this work. The standard deviations are indicated between brackets.

	CBS1146	COFT1	NRRL Y-50541
CBS1146			
COFT1	99.54 (±0.71)		
NRRL Y-50541	97.98 (±2.28)	97.89 (±2.29)	
SCRM101298	99.54 (±0.74)	99.63 (±0.66)	97.62 (±2.29)

## Supplementary Materials

The following are available online at https://www.mdpi.com/2309-608X/7/4/287/s1. Supplementary Data S1: Yeast Genome Annotation Pipeline (YGAP) annotation of the four publicly available genomes of *Torulaspora delbrueckii.* Supplementary Data S2: YGAP annotation of 19 yeast strains whose genome was not annotated (or only sparsely annotated). Supplementary Data S3: Common inferred genes in the previous and novel annotation of *T. delbrueckii* CBS 1146 that differ in the extent of the gene in the chromosome. Supplementary Data S4: Genes inferred in the current annotation of *T. delbrueckii* CBS116 that were not present in the previous annotation. Supplementary Data S5: Genes from the previous annotation of *T. delbrueckii* CBS 1146 that were not inferred in the current annotation. Supplementary Data S6: Triple-wise comparisons of the egg-NOG annotations for the three *T. delbrueckii* strains considered. Supplementary Data S7: Kyoto Encyclopedia of Genes and Genomes (KEGG) Orthology associated with the *Torulaspora delbrueckii* COFT1-predicted protein-coding sequences. Supplementary Data S8: Number of protein-coding sequences that were homologous to the *Torulaspora delbrueckii* COFT1 strain detected in 386 fungal strains. Supplementary Data S9: Core group of *Torulaspora delbrueckii* proteins conserved across the 386 fungal strains considered in the analysis presented in Figure 5. Supplementary Data S10: Detailed phylogenetic tree of 386 fungal core genomes (alignment of 204 common proteins), which is summarized in Figure 5.
